# The voice of healthcare: introducing digital decision support systems into clinical practice - a qualitative study

**DOI:** 10.1186/s12875-023-02024-6

**Published:** 2023-03-13

**Authors:** Ann Frisinger, Panagiotis Papachristou

**Affiliations:** 1 Study Programme in Medicine, Karolinska Institutet, Stockholm, Sweden; 2 Division of Family Medicine and Primary Care, Department of Neurobiology, Care Sciences and Society, Karolinska Institutet, SE-141 83 Stockholm, Sweden

**Keywords:** Clinical decision support system, Digitalization, Digital transformation, Healthcare, Primary care, Malignant melanoma

## Abstract

**Background:**

There is a need to accelerate digital transformation in healthcare to meet increasing needs and demands. The accuracy of medical digital diagnosis tools is improving. The introduction of new technology in healthcare can however be challenging and it is unclear how it should be done to reach desired results. The aim of this study was to explore perceptions and experiences of introducing new Information Technology (IT) in a primary healthcare organisation, exemplified with a Clinical Decision Support System (CDSS) for malignant melanoma.

**Methods:**

A qualitative interview-based study was performed in Region Stockholm, Sweden, with fifteen medical doctors representing three different organisational levels – primary care physician, primary healthcare centre manager, and regional manager/chief medical officer. In addition, one software provider was included. Interview data were analysed according to content analysis.

**Results:**

One central theme “Introduction of digital CDSS in primary healthcare requires a multidimensional perspective and handling” along with seven main categories and thirty-three subcategories emerged from the analysis. Digital transformation showed to be key for current healthcare providers to stay relevant and competitive. However, healthcare represents a closed community, very capable but with lack of time, fostered to be sceptical to new why change needs to bring true value and be inspired by people with medical background to motivate the powerful frontline.

**Conclusions:**

This qualitative study revealed structured information of what goes wrong and right and what needs to be considered when driving digital change in primary care organisations. The task shows to be complex and the importance of listening to the voice of healthcare is valuable for understanding the conditions that need to be fulfilled when adopting new technology into a healthcare organization. By considering the findings of this study upcoming digital transformations can improve their success-rate. The information may also be used in developing a holistic approach or framework model, adapted to primary health care, that can support and accelerate the needed digitalization in healthcare as such.

**Supplementary Information:**

The online version contains supplementary material available at 10.1186/s12875-023-02024-6.

## Introduction

The pace of digitalization is accelerated in all businesses, including healthcare. The trend is driven by economics, needs for improved effectiveness, quality, demand for new services, but also by technology itself, where innovation offers new possibilities. Covid-19 has accelerated the digital trend [1]. The advancement of digital maturity in healthcare is critical and needs to be improved [1, 2]. As we are expected to live longer, with increased healthcare costs [3], we must find ways to introduce and utilize digital technology to meet future healthcare needs.

### Primary healthcare

Primary healthcare serves as the first line care provider. It should commonly manage all basic medical treatment and care that do not require hospital resources or specialised medical skills [4]. The primary healthcare leaders balance various stakeholders’ requirements, e.g., patients care, practitioners’ workload, the boards’ high demands and budget restrictions [5].

The Swedish healthcare has a history of being well regulated, to large extent politically controlled, and mainly financed through the tax system [6–8]. Sweden is divided into regions and self-governing regional councils that are responsible for the regional healthcare [8], e.g., Region Stockholm. Each patient has the possibility to choose a healthcare provider based on their own choice [6]. There is a standardised care pathway to follow when diseases are suspected [9]. This is to ensure everyone is treated within certain time and according to the same process regardless of where in the country a patient lives or what healthcare provider is initially visited.

### Malignant melanoma

Malignant melanoma is an increasing form of skin cancer globally [10–13]. If diagnosed at an early stage, it has the potential of being completely cured or removed with a better prognosis and 5-year survival rate exceeding 90% [14]. Most melanomas are discovered by the patients or their relatives, but still a significant part are found by incident during a visit to primary care [12, 15].

Malignant melanoma can be diagnosed by anamneses and physical examination of skin lesions, looking for patterns [16], described in checklists and manual algorithms like chaos and clues [17] using a dermatoscope [12, 15]. Teledermoscopy offers additional possibility to refer skin lesions, using a smartphone-connected dermatoscope, to a dermatology specialist for further review and assessment. It has been shown that the assessment of a skin lesion has higher accuracy when reviewed by several physicians than by one doctor [15, 18]. The procedure has also decreased the time to treatment [18].

### New diagnostic methods using information technology

There is a potential to improve healthcare by adopting new technology, e.g., by use of Big Data or Artificial Intelligence (AI) [19] for analytics, leverage mobile applications and social platforms to make healthcare more available to patients [20]. New methods, supported by AI, are being developed for diagnosis of melanoma. The area of digital Clinical Decision Support Systems (CDSS) is developing, with potential to increase diagnostic accuracy and patient safety [10, 21]. Previous studies have compared AI algorithms with the performance of dermatologists [13, 20, 22] and conclude that its usage can improve overall diagnostic accuracy [23]. However, the lack of understanding how AI comes to its decision [24], the need of additional training [25], new equipment [26], and other costs [25] constitute barriers for clinical adoption of AI based CDSS.

### Digital transformation

Transformation describes the change going from current state to future state [27], executing a set of actions. There are many models describing how to drive change [28], e.g., Kotter’s change model [29], or quality improvements such as Total Quality Management [30], Lean [3, 30–32] and Lean six sigma [29, 33]. Since digital transformation is much more than adopting new IT, the actions need to consider a variety of dimensions and success factors [34–36].

When a change relies on how people act, the transformation becomes more complex and cultural aspects need to be considered. The success of the change will thus be dependent on people’s perceptions, values, cognition, and acceptance [37]. Motivation is key to successful transformation to make people engaged [28].

### Introducing new information technology in primary healthcare

During recent years there has been a major drive to utilize IT technology in healthcare, especially in primary healthcare where new actors offer web-based healthcare services [38].

Introduction of new technology in primary healthcare can however be challenging [30, 35]. According to Karuppan et al. [3] healthcare has made investments in IT but ended up with less promising results due to problems during implementation, slow adoption, and incompatible systems. Harrison et al. [39] identifies the reasons for healthcare’s failure in driving strategic changes as: lack of management attention, unclear responsibilities, budget restrictions, inefficient communication with stakeholders, shortage of skilled resources, and wrong measurements.

There is a question how new technology should be deployed and used for optimizing the result for all involved parties [1, 21, 22, 30, 35]. The field of digital transformation is emerging, and more research is needed [34]. Industry has long experience of driving change [31] and digital transformation [19]. This motivates additional research and transfer of industrial experiences to support healthcare [40]. Prior studies recommend cross-disciplinary research [41], and combined skills in healthcare, technology, and organization [42].

While new technology offers opportunity to improve healthcare, it is still unclear how healthcare can drive digital transformation in a successful way. The aim of this interview-based work, was to investigate the perceptions and thoughts of selected stakeholders from three different organisational levels in primary healthcare. The study provides consolidated insights about the holistic conditions for digital transformation and the introduction of a digital Clinical Decision Support System (CDSS) into a primary healthcare organization. Specific research questions are: what can be improved in current digital transformation efforts; what are the success factors for deploying new technology; and what is specific for healthcare?

A recently developed CDSS application for diagnosis of malignant melanoma exemplified the introduction of new technology in the study.

## Materials and methods

This study was a descriptive qualitative interview study and data analysis was based on content analysis [43]. The consolidated criteria for reporting qualitative research (COREQ-32) were used in the description of the results (Additional file ﻿1) [44].

### Study population

Semi-structured interviews were conducted with selected primary healthcare stakeholders to collect information for the study analysis.

The stakeholders within healthcare are several. Arthur [37] talks about doctors, nurses, management, operations, patients in an example of stakeholder analysis. Moriates et al. [32] mentions from a cost perspective, patients, service providers and payers.

Based on what literature suggests and the authors’ own experiences, the following stakeholders’ perspectives were chosen to be included: the service providing organization management (e.g., the manager of the primary healthcare centre); the doctors (the primary care physicians); and the management or board to which the manager of the primary healthcare centre report. The study also included a CDSS application developer (non-physician), i.e., a third party that provides the technology. Participants from both public and private primary healthcare organisations within Region Stockholm were invited by email. A purposive sampling was applied, meaning that participants identified as potential candidates for a planned clinical study of a specific CDSS application for malignant melanoma were invited. Patients were not included due to practical reasons as time limitation; however, their input is valuable and expected in a future study on the present topic.

### Conducting interviews

For the semi-structured interviews, scheduled to last for 30–45 minutes, conducted as face-to-face physical or video meetings, an interview guide was used (Additional file 2).

The interview questions were derived from the research aim and the specific research questions, according to a top-down deductive reasoning [45]. The specific research questions were: What can be improved in current digital transformation efforts?; What are the success factors for deploying new technology?; and What is specific for healthcare?

The interview questions were constructed to provide information relevant to the research questions and study aims and adapted to fit within a reasonable interview timeslot. The questions for the application provider focused on the CDSS application functionality and value proposition. For the other stakeholders, five main interview questions covered the persons’ role and organization, healthcare differentiators, digitalization, malignant melanoma diagnosis and the CDSS application for malignant melanoma. The follow-on questions were similar for the groups, but more detailed and specific for the healthcare centre leader, and even more for the doctors. The doctors were also asked about their view on patients.

The interviews, conducted by the lead author (A.F.), were held in Swedish and the individual audio-recorded answers were documented pseudo-anonymously.

### Data analysis

The transcription and analysis of the interview answers was made by the lead author (A.F.), following content analysis methodology [43]. The analysis resulted in meaning units and condensed meanings. A meaning unit is a part of a text, e.g., words, sentences, or sections, that form a context and constitute the base of the analysis. Later, both authors (A.F. and P.P.) re-iterated through the material, to validate the organization of meaning units into codes, subcategories, and main categories. One central theme was formulated. Additional file 3 describes the abstraction path, going from meaning unit to condensed meaning, code, subcategory, and main category.

SWOT (Strength, Weakness, Opportunity, Threat) analysis is commonly used when developing a business strategy. This is to understand what strengths to further leverage and weaknesses to overcome in an organisation. What opportunities are there to explore and what threats need to be mitigated [3]. This study used a SWOT matrix to provide an integrated overview of the content analysis result. Although a SWOT analysis is not required, or part of standard methodology when performing content analysis, it may provide a comprehensive framework of the result and present the application of the study results.

## Results

In total, 54 selected primary healthcare stakeholders were invited by email and sixteen accepted and consented for the semi-structured interviews. The participants were distributed as follows: five regional managers/chief medical officers, five local primary healthcare centre managers, five primary care physicians (specialists in family medicine), and one CDSS application provider representative (Additional file 4). As it turned out, all participants but the CDSS provider were medical doctors by profession. The interviews lasted on average for 43 minutes each. The transcription took 70 hours and resulted in 97 pages of text, or 6 pages per interview on average (using single row, times new roman 12 pt.)

The analysis of the transcribed interview-answers resulted in almost 600 meaning units. The ~ 600 meaning units were coded into 78 codes, then arranged into 33 subcategories, 7 main categories and 1 central theme (Additional file 5). An overview of the content analysis result is shown in Table 1.

**Table 1:** Overview of content analysis result with subcategories, main categories, and central theme

Subcategory	Main Category	Central theme
The value is experienced as low compared to the effort spent	Barriers to change in healthcare	Introduction of digital CDSS in primary healthcare requires a multidimensional perspective and handling
Not involved in the change process		
Scepticism to new		
Change resistance and lack of inspiration		
Motivate people to commit	Success factors for change in healthcare	
Healthcare culture for change		
Pilot to evaluate		
Leadership for change		
Communicate to understand		
Involve people in the frontline		
Strategy for digitalization and integration		
See it as an investment		
The governance and the organization	Healthcare differentiators	
The view of the market and the client		
The medical practitioners		
The difficulty to measure and follow up		
Specific strengths in primary healthcare		
Specific weaknesses in primary healthcare		
Specific opportunities for primary healthcare		
Specific threats to primary healthcare		
The level of IT maturity	The level of IT maturity and quality management maturity in healthcare	
The level of quality improvement maturity		
Restricted capacity	Melanoma diagnosis problems with current solution	
Safety		
The cost aspect		
Safety and validity	Challenges and prerequisites when introducing a digital CDSS (for malignant melanoma)	
Operating model		
IT security		
Product origin, ownership, and liability		
The investment		
Integrate and support the business		
Stakeholders’ commitment	Primary healthcare stakeholders’ commitment to use a digital CDSS solution	
Patients’ trust		

The central theme “Introduction of digital CDSS in primary healthcare requires a multidimensional perspective and handling” includes conditions necessary to consider for a successful digital transformation in healthcare. The analysis provided a holistic and healthcare-ified (inspired by Frisinger [46]) view of those conditions. The view includes to learn from previous mistakes, adopt success factors for change, adjust according to healthcare differentiators, consider the level of IT maturity and quality management maturity of an organization, identify specific improvement areas (e.g., related to malignant melanoma diagnosis) and reflect on new challenges and prerequisites that will appear when changing the way of working. It also encompasses the needed commitment and trust from the stakeholders involved and impacted by the change.

The following sections describe the findings from the semi-structured interviews using the main categories and subcategories as titles. Quotations from interviews have been translated to English by the lead author (A.F.). The original quotations in Swedish are shown in Additional file 6.

As a complement to the content analysis, an integrated view of the results is presented in a SWOT diagram, see Additional file 7.

### Barriers to change in healthcare

There are many reasons why previous digital transformation efforts have failed. The following represents what the interview respondents have experienced or what they believe is the reason.

#### The value is experienced as low compared to the effort spent

New IT solutions were said to consume more time and effort than the benefit those provided initially. According to many interviewees a reason for failing could be a bad technical solution not meeting the needs or not supporting the current way of working. Further, if the training takes several hours, it will create a lot of stress. If the solution does not provide obvious direct value, it will not be used.

*“The objective with this is to make things easier I say, but they are exhausted, or the workload is high, so they don’t want any new program in their computer”* (interviewee 7).

Many respondents said that there is no time to learn about new technical solutions and the new tools or systems are seen as disturbing. Some interviewees said that if the introduction is not good and well communicated there is a risk for failure.

*“High workload - to learn a new way of working in parallel is disturbing and the new tool gets unused”* (interviewee 10)

#### Not involved in the change process

Several participants said that if the solution was designed, selected, and decided by people far away from the daily operation, and lacking medical experience and knowledge about how work is performed, important requirements would be missed and end-user buy-in would be lost.

*“What is directed from above does seldom engage employees much”* (interviewee 9)

#### Scepticism to new

It was also discussed that if the solution was not invented here, healthcare professionals (doctors) might get sceptic. Proven evidence was said to be needed to convince the medical staff that it really works.

*“Doctors can be very sceptical, they only trust science and well proven experiences.”* (interviewee 4).

#### Change resistance and lack of inspiration

Someone said there is no immediate reason to change, and IT is not the right solution - it works well if just more resources or budget is added. One voice said that many people in healthcare are focused on the medical issues and unaware of what opportunities modern technology can bring – there is no source for inspiration. It was also mentioned that the digitalization in healthcare is limited by what is procured.

*“external surveillance, because I think the employees in healthcare need to see what is available”* (interviewee 10)

### Success factors for change in healthcare

In the interviews, success factors for change were discussed, i.e., conditions that should be fulfilled to perform a successful digital transformation with sustainable results.

#### Motivate people to commit

Motivation as a key element was discussed with many respondents, where two types were mentioned; Motivation by stimulation where initiatives have started from the practitioners - something they wanted to do - or an advantage that was provided for those who participated. The other alternative was described as a mandatory change and an example was given - the application for digital patient contacts that were implemented on broader base during the pandemic. Not many said “no” to that change since there were no options, and everyone understood why it was needed.

*“Important to always gain interest and engagement from the employees and adjust to what is working”* (interviewee 5)

#### Healthcare culture for change

Having the right organizational culture was also directly or indirectly highlighted as a success factor for change by several respondents. That could be about mindsets and behaviours, but also who is the messenger, who can learn from whom, how is a change communicated, if it is driven on voluntary or mandatory basis, with or without buy-in from the practitioners.

*“The important thing when introducing something new is to have the group of doctors with you”* (interviewee 16)

#### Pilot to evaluate

Many interviewees said it was better to start small scale, testing a product during a pilot. That would give time to evaluate and the experiences from the pilot could be shared.

*“Start small … the advantage of being part of a pilot is that you have the chance to influence”* (interviewee 3)

A couple of respondents mentioned examples where new IT solutions were piloted with success, even though the procurement process had stopped or delayed the deployment, without further notice.

#### Leadership for change

Leadership was discussed from a variety of perspectives and seen as important to be able to make a successful change. Leaders should take the lead, participate, get involved, be able to communicate, offer good balance between mandatory and voluntary participation, follow up on progress, as well as adjust or even stop initiatives in case the result was not satisfactory. They should also bring forward role models - clinically active were seen to have higher legitimacy.

*“Managers need to take the lead and test it”* (interviewee 3)

*“Would delegate to medical responsible doctor who works in the clinic and has higher legitimacy among the doctors, but of course support”* (interviewee 4)

#### Communicate to understand

Good communication was viewed as an important feature. To be able to clearly, timely and to the right amount describe what was the purpose and expected benefit of the initiative. Training should also be adapted in time and format to fit everyone’s busy agenda and be available in a practical way. Support should be available when needed.

*“The reason for doing this, if one can answer that question – why – we will get the employees with us”* (interviewee 4)

#### Involve people in the frontline

The initiatives should preferably come from the field. If coming from above (from top management) the interviewees said that practitioners should be involved. They said that bottom-up approaches, where medical practitioners got involved in design and decisions, should supplement top-down to capture good ideas from the field and thereby gain buy-in.

*“I think it is extremely important to involve the employees in these processes”* (interviewee 9)

*“Some colleagues, practitioners that are good in certain areas are utilized to establish new things and drive change, then there are some that are less change minded but they join in the end, and we do not stop a change because of that”* (interviewee 6)

Also, involvement of patient user groups was said to be advantageous. Initiatives coming from external, i.e., from people lacking medical skills and experiences such as consultants or IT professionals, were seen as less attractive. Combined skills were however seen as something positive, but medical skills were the key to get acceptance and trust.

#### St*rateg*y for digitalization and integration

Some interviewees mentioned that the new IT solution should be part of the overall digital strategy. It was said that there is only room for a handful lonely non-integrated applications. Further, the solution should support the business. The change needs to make daily life easier for practitioners, improve the quality, or meet patient needs and expectations.

*“Good digital system, you do not think about them, they just work”* (interviewee 16).

#### See it as an investment

Since the workload is commonly high, the interviewees said that it would be advantageous if the time spent on testing and learning could be seen as an investment and that lower performance should be allowed for a shorter period. Later, when the change was operationalized, there would be time to catch up.

“*As it is now,* some *experience that we add a tool, but we need over time to show that we can cut other things”* (interviewee 6)

*“It is OK not to have the same telephone-availability to be able to direct the traffic to the new tool - so you need for a period of time to reduce the telephone-availability and accept that there will be some complaints.”* (interviewee 9)

### Healthcare differentiators

The below summarizes what the interviewees believed differ healthcare from other industries.

#### The governance and the organization

The interviewees described healthcare as politically controlled and financed and that it was well regulated (e.g., IT security). This was described both by public as well as private interviewees.

Healthcare was said to be hierarchical (although in Sweden equalized with a consensus way of thinking), conservative, lacking continuity and a long-term perspective.

*“It is a challenge to work in politically governed organizations since the long-term perspective is lacking”* (interviewee 11)

Further, healthcare was described as slow to change, even though the pandemic served as a strong motivator to quickly complement traditional physical patient meetings with digital contacts.

Some interviewees explained that the numerous regions and private organizations working according to local processes with a variety of non-standardized systems and tools, that was both complex and fragmented, had created an environment hard to cooperate in. However, they said that since main directions is coming from above, the mission is clear for everyone – to provide good and safe patient care.

#### The view of the market and the client

The stakeholders rather talked about patients and not so much about clients, nor were they fan of talking about market, market offerings or profit (some interviewees directed the discussion to talk about the patients instead).

*“I do not like to talk about clients, we have patients. It is a wrong wording I believe; it is the reason I became a doctor”* (interviewee 6)

Many interviewees expressed a deep concern about a new type of competition, digital healthcare providers, that has recently gained market share along with healthcare budget and resources. Some said the new actors had created market share from client-demands, opposite to the client-need principle that traditional players follow. There was a fear among the respondents that the compensation system cannot balance this new scenario fast enough. The interviewees said it could create additional pressure on traditional primary healthcare providers, so they end up with complex patient cases, heavy workload, and a difficulty to retain staff. Some expressed a concern that they are competing according to unequal rules.

*“Now, it feels like other forces take over … where my experience is that these new players … deplete the traditional primary healthcare providers both on personnel and money”* (interviewee 4)

Further the interviewees described the clients in healthcare, i.e. the patients, as exposed, vulnerable, having health problems, and expecting help to be cured or to get a confirmation that they are fine.

The healthcare was said to be distributed in a fair way prioritizing the persons with the highest need, i.e., the patient is not in driver’s seat to choose freely from the services. The care needs to be agreed with the doctor.

#### The medical practitioners

The interviewees said that the practitioners in primary healthcare production, e.g., the doctors, are mainly working in the frontline. They are well educated, trained by senior colleagues (apprenticeship) and can thereafter apply for a license. The career was described as a path leading to become a practicing senior general practitioner, something which would give high legitimacy among colleagues. Further, they said they are used to work independently but still compliant to rules and regulations. The interviewees described that the practitioners are always guided by science, avoiding the risk of patient injury due to inappropriate care, and often sceptic to changes until it is proven valid by evidence. Further, they said that the practitioners are very loyal and have full focus on doing good for the patients.

*“Here you have highly educated people working in the production. In other companies, the highly educated people are working in back office with administration, management, and leadership or so.”* (interviewee 3)

*“This means there are many highly educated people used to do as they like”* (interviewee 3)

Modernization of medical school and training was discussed in the sense to enable junior skills and capacity to be valued and to make the patients more involved in the care process.

#### The difficulty to measure and follow up

The services provided by primary healthcare were said to be diverse, with a need to customize the care for each patient – i.e., making it hard to deliver in a factory like manner. Healthcare was also said to be difficult to measure and to follow up on the performance.

*“How to measure it, I do not know”* (interviewee 13)

#### Specific strengths in primary healthcare

The interviewees said that a strength in primary healthcare is that the mission is clear. They have the patient in focus and are good at conducting physical patient meetings.

*“Physical meetings – digital contacts do not add much more than a telephone contact.”* (interviewee 14)

The interviewees said they are good to manage change when it is required, well-motivated, or within comfort zone.

Further, they said the massive frontline staff is loyal, dedicated, and have high medical skills.

*“What is working well is an admirable loyalty among many employees.”* (interviewee 9)

#### Specific weaknesses in primary healthcare

The weaknesses were said to be the high workload. In context of transformation, it could mean that additional tasks such as learning new IT might get ignored.

“*Our biggest challenge is to sufficiently be able to take care of our clients.”* (interviewee 15)

It was described that the potential of IT is not fully utilized, there is a lack of digital coordination and standards, the recent shift of patient meetings into digital caused some issues. Also, a significant part of the respondents mentioned problems with the system of medical records, e.g., describing it as a large, digitized copy of a paper journal.

*“Exciting IT project can start in one part of the organization while other parts do not know about it, so it is difficult from an IT structural way to get everyone onboard the train”* (interviewee 8)

Further, the usage of resources was seen as ineffective, and the culture was said not to encourage change. Also, the procurement process could be a delaying factor for change.

#### Specific opportunities for primary healthcare

A vast majority mentioned IT and further digital transformation as an opportunity.

*“We could be more digital when we work”* (interviewee 8)

It was mentioned that increased digitalization could shift the power from doctor to patient, thus making healthcare more democratic.

#### Specific threats to primary healthcare

The major threat was described as a market paradigm shift where new digital healthcare actors gained market share. This along with a changed steering and compensation model could make it hard to continue operate as before.

*“The digital clinic that steal our patients, we experience that.”* (interviewee 2)

Some interviewees also described regulations as a barrier for private and public actors to operate on equal basis.

### The level of IT maturity and quality management maturity in healthcare

#### The level of IT maturity

The views differed when assessing the IT maturity in healthcare. A vast majority said they assess the IT maturity as low or medium and that healthcare has just started its big IT transformation journey.

*“We are quite immature I would say.”* (interviewee 11)

Healthcare was compared to other industries such as banking, travel, and media, concluding those industries have progressed much further.

“*Healthcare is far behind in all digitalization, compared to other businesses.*” (interviewee 13)

A few interviewees assessed the IT maturity as rather high, mainly based on the long usage of IT (e.g., email, system of medical records, digital evaluations of EKG, digital patient meetings), and having a high ambition in that area, but also compared to other countries where Sweden was viewed as a front player.

During the interviews there were several examples describing inefficiencies in healthcare e.g., unnecessary duplicate tests and examinations, patients wrongly booked to a primary care doctor instead of other instance or profession, inefficient or incorrect triage of patients. The reason was said to be due to not using the power of modern IT technology, inadequate use, or non-compatible systems (e.g., lack of standards and agreed interfaces). There were also several ideas on how IT technology could be used to make healthcare more efficient and relevant. Regarding existing digital strategies, the interviewees’ responses ranged from having none, one to several strategies – but this was often viewed as non-cohesive and difficult to formulate. It was mentioned that the IT budget was too low.

#### The level of quality improvement maturity

Regarding quality improvement maturity, there was as variation in the answers on how change and quality improvements was driven in the various organizations. Some said there are initiatives ongoing at all levels. Other said there is no structured way to capture ideas from the employees or there is no time to drive change.

*“Time is short, a first priority is to take care of the patients and it is hard to make time available for development work.*” (interviewee 13)

There was also a view that primary healthcare differ from other healthcare since there are so many different cases, diagnosis, treatments, which make it hard to standardize.

### Melanoma diagnosis problems with current solution

#### Restricted capacity

Several interview respondents identified a problem that skills, equipment, and capacity to examine skin lesions is limited and that they are dependent on a few specialized doctors. If volumes increase, there will not be enough dermatologists to manage the needs.

It was described that at the primary healthcare centres, there is a tendency to book patients with skin lesions to only a few doctors. Patients must then wait until those doctors are available, or they are booked to another doctor, perhaps less experienced in examining skin lesions. Patients often show their spots when they visit a doctor for another reason. If the doctor is not experienced, it is common to ask the dermatology experienced colleague for a second opinion or to get help to use a dermatoscope and send a referral. This was said to be disturbing if that happened frequently or if the doctor had another patient on the room.

*“They also knock on the door and that is a disadvantage with the system.*” (interviewee 13)

From a patient perspective, it was said that they might need to wait to get an appointment with the right doctor, and that it can be a bit hazardous of what doctor gets the appointment – it could then be needed to reschedule the patient. This could defer the diagnosis and treatment. The respondents said there is potential to improve the patient-flow in this area.

#### Safety

Some interviewees described a risk that a doctor could make a mistake in the diagnosis and a malignant skin lesion could be left unnoticed until a later stage. That could be due to doctor’s lack of experience or simply the human factor - misjudging a case.

*“It is not a 100% safe system even if there are two colleagues behind.*” (interviewee 15)

#### The cost aspect

A few respondents were worried about the cost for equipment, such as additional dermatoscopes.

*“...ideally, there should be one in every room which could easily be used to take a picture, in the future it can be that way when the costs get lower”* (interviewee 7)

Only a few mentioned economy as a problem in the context of sending referrals to the dermatology specialists for a second opinion. They explained that the cost for sending referrals is not immediately visible to the primary care centres in Region Stockholm but is covered by society as tax.

Sending referrals for second opinion could also delay the process with a couple of days. But since many primary healthcare centres in Region Stockholm have recently changed to use teledermoscopy, many stakeholders were satisfied with this new improved method. It was however mentioned that primary care centres in other parts of the country operate differently when diagnosing melanoma, i.e., there is a lack of method standardization.

### Challenges and prerequisites when introducing a digital CDSS for malignant melanoma

A CDSS application can be proposed as a solution to solve some of the issues identified with current way of diagnosing malignant melanoma. One interviewee informed there are several products being developed using artificial intelligence (AI) in combination with dermatoscope and there are examples of such applications in the UK and Sweden. The Swedish product was described as a decision support tool for medical professionals, to help in their effort to evaluate if a skin lesion is likely to be a malignant melanoma and in need of further analysis and treatment. It was said that the doctor can use an existing smartphone with an installed application, connect the phone to a dermatoscope and take a picture with the phone’s camera. The image could then be sent to a server to be analysed by an AI algorithm and directly provide an answer such as “low risk of being a melanoma” or “high risk of being a melanoma”. The application does not provide any reasoning for the given response. The doctor will thereafter decide how to proceed.

When introducing new technology, new challenges can appear. This is what the respondents identified during the interviews.

#### Safety and validity

Most of the interviewees mentioned safety and validity as a mandatory requirement for the new IT solution. In the example of malignant melanoma, the common view was that the accuracy of the tool’s assessment should at least be as good as if it was performed by a dermatologist. The sensitivity of the tool was said to be more important than the specificity if needed to prioritize, although the specificity was also said to be important to avoid unnecessary surgery.

*“Must at least be as good or bad as it is performed manually today.*” (interviewee 14)

*“It needs to be safe. And how can you build a safe system, that is a challenge.”* (interviewee 12)

The interviewees said that the product needs to be evaluated and approved/certified before final deployment. The interviewed CDSS application developer explained that for the Swedish CDSS application, a clinical trial was going to be performed with the objective to obtain a Conformité Européenne (CE) certification with a European Union Medical Device Regulation (EU MDR) marking - a standard for health and safety according to medical technical directive within Europe.

#### Operating model

Many respondents mentioned that the full healthcare process and other aspects of the operating model need to be considered and described when performing a change.

*“In terms of processes, then we need to think, should we offer spot-examinations”* (interviewee 2)

#### IT security

IT security needs to be clarified and risks analysed. Many said this is mandatory and will be a showstopper if the requirement cannot be met.

*“The other thing is that when there is small startup digital separate solution, then the data security or ownership of data is not fully in control.”* (interviewee 5)

#### Product origin, ownership, and liability

Some said there is also a need to clarify details around the product, such as who is the developer, product ownership, services delivery commitments, liabilities, terms and conditions, planned releases, and to what extent services and support will be provided.

*“Is it used elsewhere, is it developed in Sweden, you need to know more or is it just some hocus pocus thing. You need to have the background of such things.”* (interviewee 10)

The question of liability was discussed with the interviewees. A majority said that it is the doctor (or the organisation) who is responsible, a few said it is the application provider, and some pointed out there is a need to clarify this before deployment.

#### The investment

Low cost was requested by some interviewees. They realized that in the case of CDSS application, there might be a need for additional equipment as well as a licence for using the application. This cost should then be compared to today’s sending referrals for second opinion.

*“It should basically not cost anything”* (interviewee 2)

Some interviewees said the product should be user friendly and easy to learn. They requested communication packages, however not including too much information since that would take valuable time, scare practitioners away and be viewed as disturbing.

#### Integrate and support the business

Healthcare’s IT system platforms was said to be fragmented in Sweden. Some respondents mentioned this as a problem. They said that new tools and systems preferably should be integrated to avoid special handling and monitoring which could otherwise impact patient safety and create additional work. The respondents said that the solution should support the business and be part of a bigger picture.

*“There are good intentions, excellent systems, but it often fails since it is not possible to add one more system. That does not contribute to the full picture. If a system can talk to existing systems, then it becomes interesting.*” (interviewee 5)

### Primary healthcare stakeholders’ commitment to use a digital CDSS solution

#### Stakeholders’ commitment

There was a wide variation in the responses related to the stakeholder’s commitment to change, introducing a digital CDSS for malignant melanoma, also within a group of stakeholders. It was also mentioned by several respondents that the diagnosis and treatment of pigmented skin lesions is only a small part of the work that is performed at a primary care centre, there are other more frequent areas to focus such as chronic diseases (e.g., cardiovascular, diabetes), thus limiting the interest to improve this area further. Within the group of board / regional managers, they spanned from being indifferent to enthusiastic. The primary healthcare centre leaders could be seen as helpful up to enthusiastic. The doctors were opposed to helpful. Most of the interviewees said that their interest was dependent on the conditions around the product and the developer.

*“I have to be engaged. It must be me who believes in it. Otherwise, I am unable to sell it.”* (interviewee 12)

“*I am currently not very interested.*” (interviewee 13)

#### Patients’ trust

The doctors said that patients would probably accept the solution and be compliant if the procedure was presented in a comprehensive and trustful way by a doctor who supported it.

*“What the patient think? Good if I explain pedagogically the advantages, assure it is not an IT guy who believes this is exciting but a well-tested, validated method which makes us confident in the result – then I believe the patient would appreciate it.*” (interviewee 7)

## Discussion

This interview-based qualitative study investigated the holistic conditions for introducing digital Clinical Decision Support Systems (CDSS) into a primary healthcare organization through the perceptions and beliefs of selected primary healthcare stakeholders.

The study provided a holistic and healthcare-ified understanding of those conditions. The inductive content analysis of interviews with primary healthcare stakeholders resulted in the central theme “Introduction of digital CDSS in primary healthcare requires a multidimensional perspective and handling“, along with seven main categories, and thirty-three subcategories. These labels clarified what might go wrong today when deploying new technology, such as a CDSS for malignant melanoma, what potential success factors for change exist and what other conditions are specific for primary healthcare.

The study confirmed the need to accelerate digital transformation in healthcare to meet future needs and demands for care [1, 2] and the need to clarify how healthcare can do this in a successful way. The study finds that the view of primary healthcare characteristics, its IT usage, and the need to improve digital transformation efforts, is coherent considering the sources of interviews, literature, industry standards, and authors’ own experiences.

The interviews confirmed the need to improve IT and digital transformation in healthcare [1–3], e.g., the system of medical records does not leverage the power of IT, there are incompatible systems and non-standardized interfaces leading to inefficiencies and problems to communicate. Also, the test and roll-out of the successful teledermoscopy-program in Stockholm, Sweden, has lasted for 6 years and is still ongoing (which is slow on a market that advances fast).

While the study indicates that primary healthcare can learn about digital transformation from industry, industry should also learn from primary healthcare. Unlike other businesses, where it may be assumed you need to get promoted to manager to get higher status and legitimacy, the study finds that primary healthcare has succeeded in making the work towards the client (the patient) important and credible. This to such a degree that one must stay grounded on the field to make one’s voice heard. This needs to be considered when driving change in primary healthcare, to select role-models from the medical practice and change facilitators with medical understanding - or it will not have the desired effect.

The qualitative analysis performed in the study found that primary healthcare has weaknesses that need to be mitigated to enable digital transformation in this environment. There is a need to free up resources and mobilize motivation with help from active leaders and sponsors. The transformation work should be structured, include all the steps properly or there is a risk for failure and efforts spent will be efforts wasted.

To be able to drive change there is a need for motivation [28], this is an aspect shared with other industries. Primary healthcare professionals need some initial facilitation and internal as well as external collaboration [39] to get inspiration and motivation to see change as an opportunity with value-add for them and for other stakeholders.

The interviews pointed out that the new IT should support the business and that primary healthcare has a unique strength - the massive workforce with loyal, well-educated, and capable frontline workers. New opportunities should therefore be evaluated and communicated in the light of how it could further strengthen the frontline. However, the new IT does not necessarily need to support the current way of working. It might require a wider change of operation – something that could be seen as a disturbance or threat and reason to resist change [35]. A cross-scientific view and external exchange, as suggested by other studies [34, 39], could inject ideas that go beyond how things are performed today. This may be required to elevate healthcare to a level where it can meet future demands and compete on equal terms with new actors on the market [28].

When discussing stakeholders’ commitment to change to use a CDSS application for malignant melanoma, the interviewees said that melanoma is just one of many diagnoses and seen as a smaller service-article. However, sending referrals to two dermatology specialists for second opinion can, when additional primary healthcare centres join the way of working, become a high cost for society and eventually exceed the capacity. There is a dependency on a few doctors, both at the primary healthcare centre and serving as second line. When those eventually would like to go on vacation, the shop must close. This could motivate the use of a digital CDSS for malignant melanoma, provided it can be proven safe and meet all the requirements.

When comparing the different stakeholder groups, there were many similarities in their answers, probably since most of the respondents (15 out of 16) were doctors with current or recent experience from working in the clinic and with an assumed interest in IT or dermatology. However, while those higher up in the organization were more aware of the need to perform digital transformation and concerned about how to make that happen, those working in the clinic were mainly focussed on the daily transactions (patient care) and less engaged in the need of driving change.

Previous research has recommended combined skills of healthcare, technology, and organization [42]. As medical university libraries can be very medical oriented, they require researchers to search for cross scientific literature outside the perimeter. This requires skills in other disciplines to know what to look for, or it means yet another barrier for successful digitalization of healthcare.

### Strengths and limitations of the study

The study explored the holistic, cross-disciplinary conditions for digital transformation in healthcare. A holistic perspective limits the risk of missing important aspects. The cross-disciplinary perspective was declared from start since assessments can differ depending on the background of the observer. The study included a variety of stakeholder groups, opposite to other studies that had a narrower scope and focussed only on practising doctors [41, 47, 48], or only patients [13, 49]. The diversity was necessary to provide a broad, inclusive understanding of this complex field.

One can however speculate if the selection of interviewees, those that accepted to be part of the study, either were interested in IT or dermatology, or opposite took the chance to give their view. Out of the stakeholders invited to participate, there was a higher participation acceptance rate among men (47%) than women (19%). This can indicate the level of technology interest and priority if comparing the split with a technology university where most students are men [50]. The study was also limited to one country and one region, thus the result can be coloured by local situation. Since the responses in the interviews represent the first thing that comes to mind, the views can differ if this matter was discussed directly. This means, there is a risk not all aspects have been covered by the interviews. This could however also be seen as an advantage since the open discussions to a high degree reflected the interviewees’ true opinions.

A variety of literature was reviewed to cover all aspects needed for this research. Even though the literature review does not represent a systematic full coverage, no study seemed to give a full picture of the state of the art and solving or even understanding the problem. It seems that prior research mainly has been performed without cross-disciplinary coverage and lack a holistic perspective. This indicates that this work fills a white space and provides a foundation for progressing the area of healthcare digitalization.

The trustworthiness of a qualitative study can be evaluated by studying the validity, credibility, dependability, and transferability of the result [43]). Yin [45] refers to seven strategies that can reduce the risk of lacking validity of a study. This study covered four of those: 1) triangulation to compare different perspectives; 2) the lead author’s (A.F.) prior extensive and long-lasting relation to the IT-field and management that was used to develop relevant questions for the interviews, interpret the answers, identify important patterns, evaluate findings, select, and integrate perspectives; 3) the search for trends and patterns, but also lack of those in the answers; 4) the inclusion of different stakeholder groups and environments that could be compared. The study did not use quasi statistics. Quotations were used to increase the trustworthiness and validity of the resulting analysis [45]. Each respondent validated and approved their respective quotations in the results section. Data saturation was reached but additional interviews, e.g., with patients, could add value.

All interviews were performed by the lead author (A.F.) in a similar manner to optimize the credibility. The analysis and abstraction path going from meaning unit to codes, subcategories, main categories, and central theme was performed in several iterations where the result was reviewed, discussed, and validated by the second author (P.P.). The lead author (A.F.) participated in the interviews and thereby the data-collection. The analysis (e.g., the selection of meaning units) can therefore be coloured by the experience of the author [21]. The background of the authors is presented in Additional file 1.

The material and method section describes how the study was performed and the presentation of the result aimed to provide some transparency (in balance with participant integrity) to enable the reader to decide the level of transferability. Information power can be considered achieved when the aim and the research questions can be responded to, and no new information will be reached by additional data collection [51]. The interviews continued, according to plan, until the research questions could be responded to, and the research aim could be fulfilled.

### Practical applications

The result of this qualitative study can be used to better understand the barriers and facilitators to implementing digital systems in practice. The integrated result, presented in the SWOT matrix (Additional file 7), highlight areas of strength that healthcare can build on, opportunities that should be further explored, weaknesses and threats to manage or avoid. When introducing new IT technology, such as a CDSS for malignant melanoma diagnosis, this can imply: to involve the loyal, medical frontline in the transformation work; make sure they have the required time to engage; consider that important requirements such as capacity, safety and IT security are included in the solution; make people commit by having a leadership that can communicate an understanding of the value that the change would bring; start small with pilots to evaluate if the solution meets the needs, fits into overall IT strategy, and is compatible with environment; understand the market, society, patients’, and the end-users’ needs and perceptions. Decide if there is reason to continue with full deployment of the change.

The study used a CDSS for malignant melanoma as an example, it is however the lead author’s (A.F.) view that the findings, to a high extent, is healthcare and IT-technology generic and can be further developed to help healthcare accelerate the needed digital transformation. It is also the lead author’s (A.F.) view that the findings can be used to define requirements for a healthcare-ified holistic approach that can be built, based on existing industry best practices for digital transformation.

Another aspect is health equity, which is relevant to the digital transformation in healthcare [52]. For instance, if digital tools are required for contacting healthcare it could be barrier for people without technical equipment, or those that are unable to handle it. If the doctor is using a cell phone and a camera when performing an examination, it could be difficult to understand for some patients and reduce the trust in healthcare. Some people could also have a lower trust in technology as such which needs to be considered.

### Future studies

Additional work is in progress to use the findings from the present study to develop a holistic healthcare-ified approach that after test and evaluation may be used in the clinical practice. The approach is intended to meet the specific conditions for primary healthcare identified in this study and find out whether and how experiences from other industries can be reused to support healthcare driving digital transformation successfully with sustainable results.

In addition, it would be advantageous if more cross-scientific research could be performed and shared cross-industry (both ways) to inspire and drive ideas outside the box.

## Conclusions

The central theme that emerged from the study, “Introduction of digital CDSS in primary healthcare requires a multidimensional perspective and handling”, covered the specific aspects and values that need to be taken into account when introducing new technology in healthcare. The picture is complex and goes from removing obstacles for deploying new technology to enabling drivers that make a successful change reality, all while considering healthcare differentiators, the level of IT maturity and the commitment of the organization.

By considering the findings of this study, upcoming digital transformations can improve their chance of succeeding. The gathered information also provides a foundation for developing a holistic approach for introducing digital CDSS into a primary healthcare organization. If doing so, the findings in this study can help healthcare to accelerate the needed digitalization.

## Supplementary Information

**Additional file 1: A1 Table.** COREQ-32 criteria for reporting qualitative research.

**Additional file 2: A2 Table.** Interview guide questions for stakeholders (A. English version. B. Swedish version).

**Additional file 3: A3 Table.** Example of abstraction path in content analysis.

**Additional file 4: A4 Table.** Distribution of participants in the interview study.

**Additional file 5: A5 Table.** Content analysis result**.** Content analysis with 78 codes, 33 subcategories, 7 main categories, 1 central theme.

**Additional file 6: A6 Table.** Interview quotations used in the report.

**Additional file 7: A7 Table.** A SWOT matrix presenting an integrated overview of the content analysis result.

## Data Availability

The datasets used and/or analysed during the current study are available from the corresponding author on reasonable request.

## Abbreviations

AI: Artificial Intelligence
CDSS: Clinical Decision Support System
CE: Conformité Européenne
EU MDR: European Union Medical Device Regulation
IT: Information Technology
SWOT: Strength, Weakness, Opportunity, Threat
