# Supplementary material for: The voice of healthcare: introducing digital decision support systems into clinical practice - a qualitative study
Source: BMC Prim Care. 2023 Mar 13;24:67. doi: 10.1186/s12875-023-02024-6 (PMC10008705; doi:10.1186/s12875-023-02024-6)
Supplement: Supplementary file 1 — Additional file 1: A1 Table. COREQ-32 criteria for reporting qualitative research. [file 12875_2023_2024_MOESM1_ESM.docx]

**A1 Table. COREQ-32 criteria for reporting qualitative research**

| **Domain^1^** | **Criteria^1^** | **Study criteria fulfilment** |
| --- | --- | --- |
| About the research team | 1. Interviewer | The lead author (A.F.) |
|  | 2. The author’s credentials | Lead author (A.F.): M.Sc., Ph.D.  Second author (P.P.): MD, Ph.D. |
|  | 3. The authors’s occupation | Current occupation of lead author (A.F.) is medical student at Karolinska Institutet.  Current occupation of second author (P.P.) is Clinical Research Specialist at HJN Sverige AB (Cur8) and affiliated researcher at the Department of Neurobiology, Care Sciences and Society, Division of Family Medicine and Primary Care, Karolinska Institutet. |
|  | 4. The author’s gender | Lead author (A.F.): female  Second author (P.P.): male |
|  | 5. The author’s experience and training | The lead author (A.F.) has a long career in the IT industry comprising a variety of assignments and leadership positions where she has gained extensive experience in establishing, transforming, and managing large IT-services organizations. She holds a MSc in computer engineering and a Ph.D. in IT-security from the Royal Institute of Technology in Stockholm, Sweden.  The second author (P.P.) is a specialist in General Medicine and an experienced researcher. He holds a Ph.D. in medicine from the Karolinska Institute in Stockholm, Sweden. |
|  | 6. The author’s relationship with the participants | The lead author (A.F.) conducting the interviews had no relationship with the participants before the interview. The objective was to create a situation where the interviewees felt comfortable during interview. |
|  | 7. The participants’ knowledge of the interviewer | All potential interview candidates received an email invitation together with a detailed participant information sheet about the study scope and objectives. No information was provided to the participants about the lead author except she had the role of being a medical student. |
|  | 8. Interviewer characteristics | The interviewer (lead author {A.F.}) has prior experience from IT, management, and takes a cross-scientific perspective (healthcare, IT, management). |
| Study design | 9. Methodological orientation and theory | Content analysis |
|  | 10. Sampling | Purposive |
|  | 11. Method of approach | Physical or digital meeting |
|  | 12. Sample size | 16 |
|  | 13. Non-participation | 39 (estimated – some stakeholders asked colleagues about their interest to participate) |
|  | 14. Setting of data collection | In workplace / remote workplace |
|  | 15. Presence of non-participants | None |

*(ctd on next page).*

Table ctd.

|  | 16. Description of sample | Participants from both public and private primary healthcare organisations were invited with a purposive sampling. The selected primary care centres were active in Region Stockholm and potential candidates for a planned clinical study of a specific CDSS application for diagnosis of malignant melanoma. |
| --- | --- | --- |
|  | 17. Interview guide | The questions were developed by the lead author, not piloted before but confirmed during first interview. |
|  | 18. Repeat interviews | n/a |
|  | 19. Audio/visual recording | Audio recording |
|  | 20. Field notes | n/a |
|  | 21. Duration | Avg. 43 minutes per interview. |
|  | 22. Data saturation | Data saturation was discussed in the report. During the final interviews, the response-patterns started to repeat. |
|  | 23. Transcripts returned | No |
| Analysis and findings | 24. Number of data coders | Data was initially coded by the lead author (A.F.) and later validated in a workshop with the second author (P.P.). |
|  | 25. Description of coding tree | Example provided. |
|  | 26. Derivation of themes | Theme and categories were derived from the data. |
|  | 27. Software used to manage the data | Stored on personal computer, otherwise manual management. |
|  | 28. Participant checking reporting | The participants were able to provide feedback on the findings. |
|  | 29. Quotations presented | Yes, participant quotations were presented to illustrate the categories. |
|  | 30. Data and findings consistent | There was a consistency between data presented and the findings. |
|  | 31. Clarity of major themes | Theme and categories were presented in the findings. |
|  | 32. Clarity of minor themes | Some minor themes (mentioned by a minority) were presented. |

^1^COREQ-32 checklist (Tong et al., 2007).
