# Supplementary material for: The voice of healthcare: introducing digital decision support systems into clinical practice - a qualitative study
Source: BMC Prim Care. 2023 Mar 13;24:67. doi: 10.1186/s12875-023-02024-6 (PMC10008705; doi:10.1186/s12875-023-02024-6)
Supplement: Supplementary file 2 — Additional file 2: A2 Table. Interview guide questions for stakeholders (A. English version. B. Swedish version). [file 12875_2023_2024_MOESM2_ESM.docx]

# **A2 Table. Interview guide questions for stakeholders (A. English version. B. Swedish version)**

| **A. Interview questions, English version** |
| --- |
| **Interview questions for board member / manager of primary healthcare centre leaders** |
| 1. Describe shortly your role, the organization, your expectations, and the primary goals for the organization, and how those goals are followed up. What works well, what can be improved, possible challenges and possibilities. |
| 2. What is your view of what differentiates healthcare from other businesses? |
| 3. Describe the organizations usage of IT and how digitalization is driven.   1. How would you rate the IT maturity of the organization? 2. Is there a digitalization strategy? What are the main goals and milestones? 3. What areas would you like to see improved by digitalization? 4. What are the advantages and disadvantages with digitalization? 5. How is change (generally) and digitalization (specifically) driven in the organization? Does it work well? What can be improved? |
| 4. Your view of how diagnosis of malignant melanoma is performed today and how should it be performed?   1. What is a strength with that way of working? 2. What are the weaknesses? What can be improved? |
| 5. If a CDSS application (using AI) could be used in the diagnosis of malignant melanoma, what would your expectations be and how involved would you be in the introduction?   1. What would the advantages and challenges be? 2. What is your view of liability? 3. Would the introduction impact the offer to the market? 4. Would the introduction be on the meeting agenda? 5. Would the introduction be part of a larger digitalization plan? |
| **Interview questions for primary healthcare centre leader** |
| 1-4. See interview questions for board member / manager of primary healthcare centre leaders |
| 5. If a CDSS application (using AI) could be used in the diagnosis of malignant melanoma, what would your expectations be and how involved would you be in the introduction?   1. What would the advantages and challenges be? 2. What is your view of liability? 3. Would the introduction impact the offer to the market? 4. Would the introduction be on the meeting agenda? 5. Would the introduction be part of a larger digitalization plan? 6. What would be required for a successful introduction and usage? 7. Who is driving the change / who is most interested to get it implemented? 8. Would the introduction change anything in the organization? |

*(ctd on next page).*

Table ctd.

| **Interview questions for doctor** |
| --- |
| 1. Describe shortly your role and the organization. What are your main objectives and how is that followed up? In your job, what works well, what can be improved, possible opportunities and challenges? |
| 2-4. See interview questions for board member / manager of primary healthcare centre leaders |
| 5. If a CDSS application (using AI) could be used in the diagnosis of malignant melanoma, what would your expectations be and how involved would you be in the introduction?   1. What would the advantages and challenges be? 2. What is your view of liability? 3. Would the introduction impact the offer to the market? 4. Would the introduction be on the meeting agenda? 5. Would the introduction be part of a larger digitalization plan? 6. What would be required for a successful introduction and usage? 7. Who is driving the change / who is most interested to get it implemented? 8. Would the introduction change anything in the organization? 9. How much time and effort would you spend to start work according to the new method? 10. How do you think the patient would feel about the new tool? Any benefit for the patient? |
| **Interview questions for CDSS application provider** |
| 1. How does the CDSS application work, describe the functionality? How will it learn more – AI? |
| 2. Is this the first version? Are new versions planned? |
| 3. What is the pricing model (one time cost, licences, subscription, scalability)? |
| 4. Any equipment needed? Other prerequisites? |
| 5. Can / should it be connected to the system of medical records? |
| 6. Who is the intended user? What skills is needed to use it? Any education provided? |
| 7. How is it planned to be used? Is there a need for the users to change their way of working? |
| 8. How was it tested? |
| 9. Your thoughts about IT security? |
| 10. How safe is it, what is the accuracy (compared to dermatology specialists)? What about liability? |
| 11. Has it been approved to use in healthcare? In what countries? What certificates? What standards are followed? |
| 12. Has it been used before somewhere? Did you run a pilot? Experiences? |
| 13. Are there other similar products on the market – what is different? Why should the client use your product (value proposition)? |

*(ctd on next page).*

Table ctd.

| **B. Interview questions, Swedish version** |
| --- |
| **Interview questions for board member / manager of primary healthcare centre leaders** |
| 1. Beskriv i korthet din roll och organisation, de förväntningar och primära mål du ställer på organisationen. Vad fungerar bra, vad kan förbättras, ev. utmaningar och möjligheter? |
| 2. Vad är din bild av vad som särskiljer hälso-och sjukvård från annan verksamhet? |
| 3. Beskriv organisationens användande av IT och hur digitalisering bedrivs.   1. Hur skulle du beskriva organisationens mognad när det gäller användande av IT? 2. Finns det någon digitaliseringsstrategi? I så fall, vilka mål och milstolpar finns? 3. Vilka områden skulle du vilja se förbättrades genom ytterligare digitalisering? 4. Vilka är fördelarna respektive nackdelarna med digitalisering? Hur drivs förändringsarbete (generellt) och digitalisering (specifikt) inom organisationen? Fungerar det bra? Vad kan förbättras? 5. Hur skulle du beskriva organisationens mognad när det gäller användande av IT? 6. Finns det någon digitaliseringsstrategi? I så fall, vilka mål och milstolpar finns? 7. Vilka områden skulle du vilja se förbättrades genom ytterligare digitalisering? 8. Vilka är fördelarna respektive nackdelarna med digitalisering? 9. Hur drivs förändringsarbete (generellt) och digitalisering (specifikt) inom organisationen? Fungerar det bra? Vad kan förbättras? |
| 4. Vad är din syn på hur man diagnostiserar malignt melanom idag och hur man bör arbeta?   1. Vilka är fördelarna med det arbetssättet? 2. Vilka är svagheterna? Vad kan kunna förbättras? |
| 5. Om en CDSS applikation (med AI stöd) skulle kunna användas för att diagnostisera malignt melanom, vad skulle du ha för förväntningar och hur engagerad skulle du vara i introduktionen?   1. Vilka skulle fördelarna och utmaningarna vara? 2. Vad är din syn på ansvarsfrågan? 3. Skulle introduktionen påverka erbjudandet till marknaden? 4. Skulle introduktionen finnas på mötesagendan? 5. Skulle introduktionen vara del av en större digitaliseringsplan? |
| **Interview questions for primary healthcare centre leader** |
| 1-4. See intervju frågor för “board member / manager of primary healthcare centre leaders” |
| 5. Om en CDSS applikation (med AI stöd) skulle kunna användas för att diagnostisera malignt melanom, vad skulle du ha för förväntningar och hur engagerad skulle du vara i introduktionen?   1. Vilka skulle fördelarna och utmaningarna vara? 2. Vad är din syn på ansvarsfrågan? 3. Skulle introduktionen påverka erbjudandet till marknaden? 4. Skulle introduktionen finnas på mötesagendan? 5. Skulle introduktionen vara del av en större digitaliseringsplan 6. Vad skulle krävas för att få en lyckad introduktion och användning? 7. Vem driver förändringen / vem är mest intresserad av att den börjar användas? 8. Skulle introduktionen ändra något i organisationen? |

*(ctd on next page).*

Table ctd.

| **Interview questions for doctor** |
| --- |
| 1. Beskriv i korthet din roll och organisation du är del av.   1. Vilka mål ställer din chef på dig och hur följs det upp? 2. I ditt jobb, vad fungerar bra, vad kan förbättras, ev. möjligheter och utmaningar? |
| 2-4. See intervju frågor för “board member / manager of primary healthcare centre leaders” |
| 5. Om en CDSS applikation (med AI stöd) skulle kunna användas för att diagnostisera malignt melanom, vad skulle du ha för förväntningar och hur engagerad skulle du vara i introduktionen?   1. Vilka skulle fördelarna och utmaningarna vara? 2. Vad är din syn på ansvarsfrågan? 3. Skulle introduktionen påverka erbjudandet till marknaden? 4. Skulle introduktionen finnas på mötesagendan? 5. Skulle introduktionen vara del av en större digitaliseringsplan 6. Vad skulle krävas för att få en lyckad introduktion och användning? 7. Vem driver förändringen / vem är mest intresserad av att den börjar användas? 8. Skulle introduktionen ändra något i organisationen? 9. Hur mycket tid och kraft skulle du lägga ner för att arbeta enligt den nya metoden? 10. Vad tror du patienten skulle tycka om den nya lösningen? Skulle det vara till någon fördel för patienten? |
| **Interview questions for CDSS application provider** |
| 1. Hur fungerar CDSS applikationen, beskriv dess funktionalitet?  Hur kan den lära mer - AI? |
| 2. Är detta den första versionen? Är nya versioner planerade? |
| 3. Hur är den prissatt (engångs kostnad, licenser, abonnemang, skalbarhet)? |
| 4. Behövs någon utrustning? Andra förutsättningar? |
| 5. Kan / bör den kopplas till journalsystemet? |
| 6. Vem är den tilltänkte användaren? Vilken kunskap krävs för att använda den? Erbjuds någon utbildning? |
| 7. Hur är den tänkt att användas? Behöver användarna ändra sitt arbetssätt? |
| 8. Hur är den testad? |
| 9. Hur tänker ni kring IT säkerhet? |
| 10. Hur säker är den, hur hög noggrannhet har den (jämfört med dermatolog/specialist)? Hur ser ni på ansvarsfrågan? |
| 11. Är den godkänd att använda inom vården?  I vilka länder? Vilka certifieringar finns? Vilka standards följs? |
| 12. Har den använts förut någonstans? Har ni kört någon pilot? Erfarenheter? |
| 13. Finns det andra liknande produkter på marknaden, vad är skillnaden? Varför ska kunden börja använda er produkt (värdeerbjudande)? |
