# Supplementary material for: The voice of healthcare: introducing digital decision support systems into clinical practice - a qualitative study
Source: BMC Prim Care. 2023 Mar 13;24:67. doi: 10.1186/s12875-023-02024-6 (PMC10008705; doi:10.1186/s12875-023-02024-6)
Supplement: Supplementary file 3 — Additional file 3: A3 Table. Example of abstraction path in content analysis. [file 12875_2023_2024_MOESM3_ESM.docx]

**A3 Table. Example of abstraction path in content analysis**

| **Meaning unit** | **Condensed meaning** | **Code** | **Subcategory** | **Main category** |
| --- | --- | --- | --- | --- |
| “High workload - to learn a new way of working in parallel is disturbing and the new tool gets unused” (interviewee 10) | High workload makes new tools unused | No time to learn and use – it is just disturbing | The value is experienced as low compared to the effort spent | Barriers to change in healthcare |
| “Important to always gain interest and engagement from the employees and adjust to what is working” (interviewee 5) | Engage and motivate people to change the way of working | Motivation due to a clear benefit and common interest | Motivate people to commit | Success factors for change in healthcare |
| “They also knock on the door and that is a disadvantage with the system” (interviewee 13) | Clinical work disturbance due to limited skills and capacity | Dependent on a few doctors | Restricted capacity | Melanoma diagnosis problems with current solution |
