# Supplementary material for: The voice of healthcare: introducing digital decision support systems into clinical practice - a qualitative study
Source: BMC Prim Care. 2023 Mar 13;24:67. doi: 10.1186/s12875-023-02024-6 (PMC10008705; doi:10.1186/s12875-023-02024-6)
Supplement: Supplementary file 4 — Additional file 4: A4 Table. Distribution of participants in the interview study. [file 12875_2023_2024_MOESM4_ESM.docx]

**A4 Table. Distribution of participants in the interview study**

|  | Participants, tot. | Participants, female | Participants, male |
| --- | --- | --- | --- |
| Medical doctor (primary care physician) | 5 | 3 | 2 |
| Primary healthcare centre manager | 5 | 2 | 3 |
| Regional manager/CMO | 5 | 2 | 3 |
| CDSS application provider | 1 | 0 | 1 |
| Total | 16 | 7 | 9 |
