# Supplementary material for: The voice of healthcare: introducing digital decision support systems into clinical practice - a qualitative study
Source: BMC Prim Care. 2023 Mar 13;24:67. doi: 10.1186/s12875-023-02024-6 (PMC10008705; doi:10.1186/s12875-023-02024-6)
Supplement: Supplementary file 5 — Additional file 5: A5 Table. Content analysis result. Content analysis with 78 codes, 33 subcategories, 7 main categories, 1 central theme. [file 12875_2023_2024_MOESM5_ESM.docx]

# **A5 Table. Content analysis result**

**Content analysis with 78 codes, 33 subcategories, 7 main categories, 1 central theme.**

| **Code** | **Subcategory** | **Main category** | **Central theme** |
| --- | --- | --- | --- |
| No time to learn and use – it is just disturbing | The value is experienced as low compared to the effort spent | Barriers to change in healthcare | Introduction of digital CDSS in primary healthcare requires a multidimensional perspective and handling |
| Bad solution - not meeting the needs or not supporting current way of working |  |  |  |
| Bad communication and introduction |  |  |  |
| Someone else decided – we were not involved | Not involved in the change process |  |  |
| Not invented here – sceptic to new | Scepticism to new |  |  |
| Procurement process is delaying successful pilots to be deployed | Change resistance and lack of inspiration |  |  |
| No reason to change, it works well if more budget is added |  |  |  |
| Lack inspiration |  |  |  |
| Motivation due to a clear benefit and common interest | Motivate people to commit | Success factors for change in healthcare |  |
| Motivation due to forced need |  |  |  |
| A clinical culture driving change | Healthcare culture for change |  |  |
| Need to run a pilot | Pilot to evaluate |  |  |
| Leadership and role model | Leadership for change |  |  |
| Communication and support | Communicate to understand |  |  |
| Share success stories |  |  |  |
| Involve the people/end users | Involve people in the frontline |  |  |
| A solution that supports the business | Strategy for digitalization and integration |  |  |
| Digital strategy alignment |  |  |  |
| Use of combined skills |  |  |  |
| See it as an investment to drive future value | See it as an investment |  |  |
| Political governance | The governance and the organization | Healthcare differentiators |  |
| High IT security (as an example of rules and regulations to consider) |  |  |  |
| Primary healthcare does not work like a factory |  |  |  |
| Culture in healthcare slow and conservative |  |  |  |

*(ctd on next page).*

Table ctd.

| Experienced as non-commercial / not market driven | The view of the market and the client |  |  |
| --- | --- | --- | --- |
| Patient satisfaction and care in focus |  |  |  |
| Provides services commissioned by someone else |  |  |  |
| Service is prioritized based on the need |  |  |  |
| Patient is vulnerable and dependent on doctor |  |  |  |
| Modernize doctor’s role | The medical practitioners |  |  |
| Trust scientific study evidence only (or follow senior doctor) |  |  |  |
| Well educated, loyal, dedicated practitioners in the frontline |  |  |  |
| Difficult to measure health quality | The difficulty to measure and follow up |  |  |
| Patient focus and physical meetings is a strength | Specific strengths in primary healthcare |  |  |
| Continuity with patient is a strength |  |  |  |
| Clear mission is a strength |  |  |  |
| Change under pressure or within comfort zone is a strength |  |  |  |
| Loyal staff, high medical skills in frontline is a strength |  |  |  |
| High workload is a weakness | Specific weaknesses in primary healthcare |  |  |
| Digital / IT not fully utilized is a weakness |  |  |  |
| Incompatible systems, lack of coordination is a weakness |  |  |  |
| Digital patient meeting experiences, a weakness |  |  |  |
| System of medical records is a weakness |  |  |  |
| Ineffectiveness is a weakness |  |  |  |
| Diverse and incompatible IT systems |  |  |  |
| Digitalization is an opportunity | Specific healthcare opportunities for primary healthcare |  |  |
| Patient empowerment through digitalization is an opportunity |  |  |  |
| Digital actors - a threat | Specific threats to primary healthcare |  |  |
| Compensation model and governance – a threat |  |  |  |
| Unequal rules private vs public – a threat |  |  |  |
| Less power to the doctor through digitalization – a threat |  |  |  |

*(ctd on next page).*

Table ctd.

| IT maturity Low | The level of IT maturity | The level of IT maturity and quality management maturity in healthcare |  |
| --- | --- | --- | --- |
| IT maturity Medium |  |  |  |
| IT maturity High |  |  |  |
| Quality improvement maturity Low | The level of quality improvement maturity |  |  |
| Quality improvement maturity Medium |  |  |  |
| Limited capacity for dermatological evaluation | Restricted capacity | Melanoma diagnosis problems with current solution |  |
| Dependent on a few doctors |  |  |  |
| Process / patient-flow can be improved |  |  |  |
| Present system not 100% safe and accurate | Safety |  |  |
| Cost for equipment | The cost aspect |  |  |
| Cost for sending referrals |  |  |  |
| Not standardized process |  |  |  |
| Validated and safe medical device with high accuracy | Safety and validity | Challenges and prerequisites when introducing a digital CDSS for malignant melanoma |  |
| Tested in a pilot and certified |  |  |  |
| Impact on operating model with AI decision | Operating model |  |  |
| IT security of a mobile application | IT security |  |  |
| Product ownership and services delivery commitments | Product origin, ownership, and liability |  |  |
| Legal aspect is clear (e.g., liability) |  |  |  |
| Introduction package is necessary | The investment |  |  |
| Affordable |  |  |  |
| Easy to use | Integrate and support the business |  |  |
| Integrated to current systems |  |  |  |
| Support business and strategy |  |  |  |
| Commitment from the board/ regional managers: enthusiastic-indifferent | Stakeholders’ commitment | Primary healthcare stakeholders’ commitment to use a digital CDSS solution |  |
| Commitment from the Primary healthcare leaders: enthusiastic – helpful |  |  |  |
| Commitment from the doctors: helpful - opposed |  |  |  |
| Patients trust and compliance to technology | Patients’ trust |  |  |
