# Supplementary material for: The voice of healthcare: introducing digital decision support systems into clinical practice - a qualitative study
Source: BMC Prim Care. 2023 Mar 13;24:67. doi: 10.1186/s12875-023-02024-6 (PMC10008705; doi:10.1186/s12875-023-02024-6)
Supplement: Supplementary file 6 — Additional file 6: A6 Table. Interview quotations used in the report. [file 12875_2023_2024_MOESM6_ESM.docx]

**A6 Table. Interview quotations used in the report**

| **Section** | **Quotation in Swedish**  **(original from interview)** | **Quotation in English (translated by the lead author {A.F})** |
| --- | --- | --- |
| The value is experienced as low compared to the effort spent | ”Det här är till för att förenkla säger jag men dom är utmattade eller har så mycket ändå så dom vill inte ha något nytt program i sin dator” (interviewee 7) | “The objective with this is to make things easier I say, but they are exhausted, or the workload is high, so they don’t want any new program in their computer” (interviewee 7) |
|  | ”Höga produktionskrav - att lära nytt arbetssätt parallellt blir då störande och nya verktyg blir oanvänt” (interviewee 10) | ”High workload - to learn a new way of working in parallel is disturbing and the new tool gets unused” (interviewee 10) |
| Not involved in the change process | ”Sånt som är sagt uppifrån på något vis det väcker ju sällan något stekhett engagemang hos medabetarna.” (interviewee 9) | ”What is directed from above does seldom engage employees much” (interviewee 9) |
| Scepticism to new | ”Doktorer kan vara väldigt skeptiska, man förlitar sig på vetenskap och beprövad erfarenhet.” (interviewee 4) | ”Doctors can be very sceptical, they only trust science and well proven experiences.” (interviewee 4) |
| Change resistance and lack of inspiration | ”omvärldsbevakning för jag tänkte att vårdens medarbetare behöver få se vad finns det” (interviewee 10) | ”external surveillance, because I think the employees in healthcare need to see what is available” (interviewee 10) |
| Motivate people to commit | “Viktigt att hela tiden fånga medarbetares intresse och engagemang och vara villig att justera utifrån vad som fungerar” (interviewee 5) | “Important to always gain interest and engagement from the employees and adjust to what is working” (interviewee 5) |
| Healthcare culture for change | “Det viktiga när man inför nya saker är att ha med sig läkargruppen” (interviewee 16) | “The important thing when introducing something new is to have the group of doctors with you” (interviewee 16) |
| Pilot to evaluate | Börja i det lilla … fördelen att vara med i en pilot är att man får vara med och påverka. (interviewee 3) | “Start small … the advantage of being part of a pilot is that you have the chance to influence” (interviewee 3) |
| Leadership for change | “Chefer måste gå före och prova” (interviewee 3) | “Managers need to take the lead and test it” (interviewee 3) |
|  | ”Skulle delegera till medicinskt ansvarig läkare som jobbar kliniskt och har större legitimitet i läkargruppen, men förstås stödja” (interviewee 4) | “Would delegate to medical responsible doctor who works in the clinic and has higher legitimacy among the doctors, but of course support” (interviewee 4) |
| Communicate to understand | ”Varför vi måste göra det här, om man kan svara på den frågan – varför – så kommer vi få personalen med oss” (interviewee 4) | “The reason for doing this, if one can answer that question – why – we will get the employees with us” (interviewee 4) |

*(ctd on next page).*

Table ctd.

| Involve people in the frontline | Jag tror att det är extremt viktigt att man involverar medarbetarna i dom här processerna (inteviewee 9) | ”I think it is extremely important to involve the employees in these processes” (interviewee 9) |
| --- | --- | --- |
|  | “Vissa kollegor, medarbetare som är duktiga på saker använder vi för att etablera och driva på en förändring, sedan är det några som är mindre förändringsbenägna men dom hänger med i slutändan, så vi stoppar inget förändringsarbete på grund av det” (interviewee 6) | “Some colleagues, practitioners that are good in certain areas are utilized to establish new things and drive change, then there are some that are less change minded but they join in the end, and we do not stop a change because of that” (interviewee 6) |
| Strategy for digitalization and integration | ”Bra digitala system dom tänker man inte på dom bara funkar” (interviewee 16) | ”Good digital system, you do not think about them, they just work” (interviewee 16) |
| See it as an investment | ”Nu upplevs det för en del att man lägger till ett verktyg men vi måste på sikt bevisa att vi kan skära bort andra saker” (interviewee 6) | ”As it is now, some experience that we add a tool, but we need over time to show that we can cut other things” (interviewee 6) |
|  | ”Det är OK att inte ha samma telefontillgänglighet för att ni ska kunna styra trafik mot det nya verktyget så måste ni för en period gå ner i teleforntillgänglighet och acceptera att det kommer in en del klagomål” (interviewee 9) | “It is OK not to have the same telephone-availability to be able to direct the traffic to the new tool - so you need for a period of time to reduce the telephone-availability and accept that there will be some complaints.” (interviewee 9) |
| The governance and the organization | ”En utmaning att verka i politiskt styrda organisationer för det saknas långsiktighet” (interviewee 11) | “It is a challenge to work in politically governed organizations since the long-term perspective is lacking” (interviewee 11) |
| The view of the market and the client | ”Jag tycker inte om att prata om kunder, vi har patienter. Det är ett felaktigt ord tycker jag, det ändå därför jag valt att bli läkare” (interviewee 6) | “I do not like to talk about clients, we have patients. It is a wrong wording I believe; it is the reason I became a doctor” (interviewee 6) |
|  | Nu känns det som att andra krafter tar över … och där min upplevelse är att dom här som kommit in, flera stycken nu ... utarmar dom traditionella vårdcentralerna både på personal och pengar. (interviewee 4) | ”Now, it feels like other forces take over … where my experience is that these new players … deplete the traditional primary healthcare providers both on personnel and money” (interviewee 4) |

*(ctd on next page).*

Table ctd.

| The medical practitioners | ”Här har du högt utbildade människor som arbetar verkligen ute i produktionen. I de flesta andra företag så sitter dom högst utbildade människorna inne på kontoren och jobbar liksom administrativt med styrning och ledning eller med projekt eller så.” (interviewee 3) | “Here you have highly educated people working in the production. In other companies, the highly educated people are working in back office with administration, management, and leadership or so.” (interviewee 3) |
| --- | --- | --- |
|  | “Det gör ju att det är väldigt många människor med hög utbildning som är vana att få göra som dom själva vill” (interviewee 3) | “This means there are many highly educated people used to do as they like” (interviewee 3) |
| The difficulty to measure and follow up | ”Hur man mäter det, det vet jag inte” (interviewee 13) | “How to measure it, I do not know” (interviewee 13) |
| Specific strengths in primary healthcare | ”Fysiskt besök – digitala kontakter tillför inte så mycket mer än en telefonkontakt.” (interviewee 14) | “Physical meetings – digital contacts do not add much more than a telephone contact.” (interviewee 14) |
|  | “Det som fungerar väl är en beundransvärd lojalitet som finns hos många medarbetare.” (interviewee 9) | “What is working well is an admirable loyalty among many employees.” (interviewee 9) |
| Specific weaknesses in primary healthcare | “Vår största utmaning är att räcka till för våra patienter.” (interviewee 15) | “Our biggest challenge is to sufficiently be able to take care of our clients.” (interviewee 15) |
|  | “Spännande IT projekt kan starta i en del av organisationen utan att den andra delen får reda på det så det är svårt IT stukturmässigt att få med alla på tåget” (interviewee 8) | “Exciting IT project can start in one part of the organization while other parts do not know about it, so it is difficult from an IT structural way to get everyone onboard the train” (interviewee 8) |
| Specific opportunities for primary healthcare | ”Vi skulle kunna jobba mycket mer digitalt” (interviewee 8) | “We could be more digital when we work” (interviewee 8) |
| Specific threats to primary healthcare | ”Den digitala mottagningen som snor patienter från oss, det ser vi ju.” (interviewee 2) | “The digital clinic that steal our patients, we experience that.” (interviewee 2) |
| The level of IT maturity | ”Vi är relativt omogna skulle jag säga.” (interviewee 11) | “We are quite immature I would say.” (interviewee 11) |
|  | ”Sjukvården ligger väldigt långt efter i all digitalisering, jämfört med andra branscher.” (interviewee 13) | “Healthcare is far behind in all digitalization, compared to other businesses.” (interviewee 13) |

*(ctd on next page).*

Table ctd.

| The level of quality improvement maturity | ”Tiden är ju bristvaran där, man måste i första hand sköta sina patienter och det är knappt tiden räcker till det så det är svårt med utvecklingsarbeten.” (interviewee 13) | “Time is short, a first priority is to take care of the patients and it is hard to make time available for development work.” (interviewee 13) |
| --- | --- | --- |
| Restricted capacity | ”Dom knackar på dessutom och det är väl lite nackdelen med systemet.” (interviewee 13) | “They also knock on the door and that is a disadvantage with the system.” (interviewee 13) |
| Safety | ”Det är ju inte ett 100% säkert system även om det är två kollegor som sitter bakom.” (interviewee 15) | “It is not a 100% safe system even if there are two colleagues behind.” (interviewee 15) |
| The cost aspect | ”idealt skulle man ha ett på varje rum som man lätt kan ta fram och fota av, i en framtid kan det bli så när kostnaderna sjunker (interviewee 7) | “ideally, there should be one in every room which could easily be used to take a picture, in the future it can be that way when the costs get lower” (interviewee 7) |
| Safety and validity | ”Måste vara minst lika bra eller dåligt som dom som gör det manuellt idag.” (interviewee 14) | “Must at least be as good or bad as it is performed manually today.” (interviewee 14) |
|  | ”Alltså det måste ju vara säkert. Och hur man bygger upp ett säkert system, ja det är en utmaning.” (interviewee 12) | “It needs to be safe. And how can you build a safe system, that is a challenge.” (interviewee 12) |
| Operating model | ”Processmässigt, då måste vi tänka till, ska vi då ha en prickmottagning” (Interviewee 2) | “In terms of processes, then we need to think, should we offer spot-examinations” (interviewee 2) |
| IT security | ”Det andra är när det är små startup digitala separata lösningar så har man inte alltid koll på datasäkerhetsfrågorna eller ägandet av data.” (interviewee 5) | “The other thing is that when there is small startup digital separate solution, then the data security or ownership of data is not fully in control.” (interviewee 5) |
| Product origin, ownership, and liability | ”Skulle den vara använd någon annanstans, är den forskad i Sverige, man behöver veta mer eller är det bara någon hokus pokus grej. Man behöver veta mer kring bakgrunden och sådana saker.” (interviewee 10) | “Is it used elsewhere, is it developed in Sweden, you need to know more or is it just some hocus pocus thing. You need to have the background of such things.” (interviewee 10) |
| The investment | ”Den ska egentligen inte kosta något.” (interviewee 2) | “It should basically not cost anything”(interviewee 2) |

*(ctd on next page).*

Table ctd.

| Integrate and support the business | ”Man har goda intentioner, bra ideer, utmärkta system, men ofta faller det på att vi kan inte stoppa in ett system till. Det gynnar inte helheten. När system kan prata med dom system vi har då är det aktuellt.” (interviewee 5) | “There are good intentions, excellent systems, but it often fails since it is not possible to add one more system. That does not contribute to the full picture. If a system can talk to existing systems, then it becomes interesting.” (interviewee 5) |
| --- | --- | --- |
| Stakeholders’ commitment | ”Jag måste ju vara engagerad. Det måste vara jag som tror på det. För annars kommer jag inte kunna sälja in det.” (interviewee 12) | “I have to be engaged. It must be me who believes in it. Otherwise, I am unable to sell it.” (interviewee 12) |
|  | ”I nuläget skulle jag vara lite sval.” (interviewee 13) | “I am currently not very interested.” (interviewee 13) |
| Patients’ trust | ”Vad patienten tycker? Bra om jag förklarar pedagogiskt fördelar, försäkrar om att det här är ingen IT kille som tycker att det verkar spännande utan det är en utprövad validerad metod som gör oss träffsäkrare – då tror jag patienterna skulle uppskatta det.” (interviewee 7) | ”What the patient think? Good if I explain pedagogically the advantages, assure it is not an IT guy who believes this is exciting but a well-tested, validated method which makes us confident in the result – then I believe the patient would appreciate it.” (interviewee 7) |
