# Supplementary material for: The voice of healthcare: introducing digital decision support systems into clinical practice - a qualitative study
Source: BMC Prim Care. 2023 Mar 13;24:67. doi: 10.1186/s12875-023-02024-6 (PMC10008705; doi:10.1186/s12875-023-02024-6)
Supplement: Supplementary file 7 — Additional file 7: A7 Table. A SWOT matrix presenting an integrated overview of the content analysis result. [file 12875_2023_2024_MOESM7_ESM.docx]

**A7 Table. A SWOT matrix presenting an integrated overview of the content analysis result. The mapping of categories into the matrix was made using: healthcare differentiators as strengths, weaknesses, opportunities, and threats - based on lead authors’ (A.F.) interpretation; barriers to change, the maturity level (IT security or quality management), and melanoma diagnosis problems with current solution as weaknesses; success factors and stakeholders’ commitment as opportunities; challenges and prerequisites when introducing a digital CDSS as threats (can be seen as challenges).**

| **Strengths**    + Loyal Staff, high medical skills in frontline^1b^  + Change under pressure or within comfort zone^1b^  + Clear mission^1b^  + Continuity with patient^1b^  + Patient focus and physical meetings^1b^ | **Weaknesses**    − The value is experienced as low compared to the effort spent^2^  − Not involved in the change process^2^  − Scepticism to new^2^  − Change resistance and lack of inspiration^2^  − High workload^1b^  − Digital / IT not fully utilized^1b^  − Incompatible systems, lack of coordination^1b^  − Digital patient meeting experiences ^1b^  − System of medical records ^1b^  − Ineffectiveness ^1b^  − Diverse and incompatible IT systems^1b^  − The governance and the organization^1a^  − The view of the market and the client^1a^  − The difficulty to measure and follow up^1a^  − Closed community^1c^  − The level of IT maturity^3^  − The level of quality improvement maturity^3^  − Restricted capacity^4^  − Safety^4^  − The cost aspect^4^ |
| --- | --- |
| **Opportunities**    + Motivate people to commit^5^  + Healthcare culture for change^5^  + Pilot to evaluate^5^  + Leadership for change^5^  + Communicate to understand^5^  + Involve people in the frontline^5^  + Strategy for digitalization and integration^5^  + See it as an investment^5^  + Digitalization^1b^  + Patient empowerment through digitalization^1b^  + The medical practitioners^1a^  + Stakeholders’ commitment^6^  + Patients’ trust^6^ | **Threats (or challenges)**    − Digital actors (on market)^1b^  − Compensation models and governance^1b^  − Unequal rules private vs public^1b^  − Less power to the doctor through digitalization^1b^  − Safety and validity ^7^  − Operating model ^7^  − IT security ^7^  − Product origin, ownership, and liability ^7^  − The investment ^7^  − Integrate and support the business ^7^ |

^1-7^ Main categories: ^1a^ Healthcare differentiators; ^1b^ Healthcare differentiators from specific strengths, weaknesses, opportunities or threats in primary healthcare – using codes (level below subcategory); ^1c^ Healthcare differentiator added by lead author (A.F.) since interviews found that primary healthcare professionals form a closed community where medical skills score high; ^2^ Barriers to change; ^3^ The maturity level (IT security or quality management); ^4^ Melanoma diagnosis related; ^5^ Success factor for change; ^6^ Stakeholders’ commitment; ^7^ Challenges and prerequisites when introducing a digital CDSS
